# Fast Optical Investigation of Cardiac Electrophysiology by Parallel Detection in Multiwell Plates

**DOI:** 10.3389/fphys.2021.692496

**Published:** 2021-09-03

**Authors:** Caterina Credi, Valentina Balducci, U. Munagala, C. Cianca, S. Bigiarini, Antoine A. F. de Vries, Leslie M. Loew, Francesco S. Pavone, Elisabetta Cerbai, Laura Sartiani, Leonardo Sacconi

**Affiliations:** ^1^European Laboratory for Non-linear Spectroscopy, Sesto Fiorentino, Italy; ^2^National Institute of Optics, National Research Council, Florence, Italy; ^3^Department of Neurosciences, Psychology, Drugs and Child Health, University of Florence, Florence, Italy; ^4^Core Research Laboratory, ISPRO, Florence, Italy; ^5^Laboratory of Experimental Cardiology, Department of Cardiology, Leiden University Medical Center, Leiden, Netherlands; ^6^R. D. Berlin Center for Cell Analysis and Modeling, University of Connecticut School of Medicine, Farmington, CT, United States; ^7^Department of Physics and Astronomy, University of Florence, Sesto Fiorentino, Italy

**Keywords:** optogenetics, voltage imaging, action potential, multiwell plate, microscopy

## Abstract

Current techniques for fast characterization of cardiac electrophysiology employ optical technologies to control and monitor action potential features of single cells or cellular monolayers placed in multiwell plates. High-speed investigation capacities are commonly achieved by serially analyzing well after well employing fully automated fluorescence microscopes. Here, we describe an alternative cost-effective optical approach (MULTIPLE) that exploits high-power LED arrays to globally illuminate a culture plate and an sCMOS sensor for parallel detection of the fluorescence coming from multiple wells. MULTIPLE combines optical detection of action potentials using a red-shifted voltage-sensitive fluorescent dye (di-4-ANBDQPQ) with optical stimulation, employing optogenetic actuators, to ensure excitation of cardiomyocytes at constant rates. MULTIPLE was first characterized in terms of interwell uniformity of the illumination intensity and optical detection performance. Then, it was applied for probing action potential features in HL-1 cells (i.e., mouse atrial myocyte-like cells) stably expressing the blue light-activatable cation channel CheRiff. Under proper stimulation conditions, we were able to accurately measure action potential dynamics across a 24-well plate with variability across the whole plate of the order of 10%. The capability of MULTIPLE to detect action potential changes across a 24-well plate was demonstrated employing the selective K_*v*_11.1 channel blocker (E-4031), in a dose titration experiment. Finally, action potential recordings were performed in spontaneous beating human induced pluripotent stem cell derived cardiomyocytes following pharmacological manipulation of their beating frequency. We believe that the simplicity of the presented optical scheme represents a valid complement to sophisticated and expensive state-of-the-art optical systems for high-throughput cardiac electrophysiological investigations.

## Introduction

Fast investigation of cardiac electrophysiology is found convenient for the assessment of cardiac ion channel activity of new chemical entities (Dunlop et al., 2008). In this respect, optical techniques for actuating and sensing cardiac action potentials (APs) represent a reference method for preclinical drug screening and cardiotoxicity testing, especially in combination with human induced pluripotent stem cell-derived cardiomyocytes (hiPSC-CMs) (Klimas et al., 2016). Conventional methods for multisite optical interrogation generally require movement of the sample (McGlynn et al., 2018) or of the imaging system (Hansen et al., 2010). These approaches intrinsically limit the acquisition time and require complex and expensive fully automated microscope platforms. Recently, Ashraf et al. (2021) proposed an innovative optical scheme (Random Access Parallel; RAP) that enables near-simultaneous imaging of multiple sites without moving parts or robotics. Although the system was successfully applied for imaging the contraction waves of multiple cardiac monolayers, the possibility of performing high-speed voltage imaging using a fluorescent voltage-sensitive dye (VSD) is precluded due to the requirement of coherent light in the RAP image formation mechanism.

Here, we describe a simple and cost-effective optical approach (MULTIPLE) that exploits high-power light-emitting diode (LED) arrays to globally illuminate a multiwell plate and an sCMOS sensor coupled with a camera lens for parallel detection of the fluorescence coming from multiple sites. The platform has been tested and characterized using VSD-loaded HL-1 cells (i.e., mouse atrial myocyte-like cells) stably expressing the blue light-activatable ion channel CheRiff (Hochbaum et al., 2014) and spontaneously beating hiPSC-CMs. MULTIPLE optically stimulates channelrhodopsin-expressing HL-1 cells across the multiwell plate with blue light pulses assuring constant pacing rates (Bruegmann et al., 2010) while global plate illumination with red light allows recording of APs using the near-infrared VSD di-4-ANBDQPQ (Matiukas et al., 2007). Notably, the use of a red-shifted VSD avoids ChR2/CheRiff excitation allowing optical recording without introducing any variation of resting membrane potential (Scardigli et al., 2018). The capability of MULTIPLE to detect electrophysiological changes was demonstrated in drug dose titration experiments in HL-1 cells and in hiPSC-CMs.

## Materials and Methods

### HL-1 Cell Culture and Staining

HL-1 cells were obtained from Sigma-Aldrich (LOT: 2955183) and were seeded on gelatin-fibronectin (Sigma-Aldrich, Schnelldorf, Germany)-coated 25-cm^2^ culture flasks, as previously described (Claycomb et al., 1998; Sartiani et al., 2002a). Briefly, cells were maintained in ‘Claycomb Medium’ (JRH Biosciences, Lenexa, KS), supplemented with 10% fetal bovine serum (Life Technologies, Cergy Pontoise, France), 4 mM L-glutamine (Life Technologies), 10 μM noradrenaline (norepinephrine; Sigma-Aldrich), and 1× penicillin-streptomycin (Life Technologies). Cultures were maintained at 37°C, in an atmosphere of 5% CO_2_ and 95% air at a relative humidity of ≈ 95%; medium was changed every 24 h. HL-1 cells were split at 100% confluency, using a 5-min enzymatic dissociation with trypsin-EDTA (Life Technologies). The enzymatic reaction was stopped by adding medium and the sedimented cells were either replated at a split ratio of 1:3 to maintain cell culture or 1:4 for transduction and staining. Before recordings, total live cell counts were determined after enzymatic dissociation by Trypan blue exclusion assay using a LUNA-II automated cell counter (Logos Biosystems, Anyang, South Korea). Voltage imaging was performed in Tyrode’s solution (mM): D-(++)-glucose 10, NaCl 140, KCl 5.4, CaCl_2_ 1.8, MgCl_2_ 1.2, HEPES 5.0, adjusted to pH 7.3 with NaOH and supplemented with 4 μg/mL of di-4-ANBDQPQ (Matiukas et al., 2007). E-4031 (Sigma-Aldrich) stock solution (10 mM) was prepared in H_2_O and diluted in Tyrode’s solution to 10 μM.

### Transduction With Viral Vectors

In order to generate a channelrhodopsin-expressing cell line, HL-1 cells were plated on precoated 6-well plates. At 70–80% confluency, cells were incubated for 24 h at 37°C in 5% CO_2_-95% air with channelrhodopsin-encoding adeno-associated virus vector (AAVV) or lentiviral vector (LV) particles. The AAVV particles were prepared from shuttle plasmid pAAV.CAG.hChR2(H134R)-mCherry.WPRE.SV40 (Addgene, 100054-AAV9), which codes for the *Chlamydomonas reinhardtii* channelrhodopsin2 (ChR2) gain-of-function mutant H134R extended at its C-terminus with the fluorescent protein tag mCherry. The LV particles were custom-made using shuttle plasmid LV.GgTnnt2.CheRiff∼eGFP.WHVPRE, which codes for a C-terminally enhanced green fluorescent protein (eGFP)-tagged version of the *Scherffelia dubia* channelrhodopsin. To improve transduction efficiency, the inocula contained 8 μg/mL of the cationic polymer polybrene (Sigma-Aldrich) in order to neutralize repulsion between virions and the cell surface.

### Flow Cytometry

Flow cytometry was performed using a 13-color, 4-laser CytoFLEX S N-V-B-R flow cytometer, equipped with 405-, 488-, 561-, and 638-nm lasers (B78557, Beckman Coulter, Brea, CA, United States) and operated by CytExpert Software v1.2 (Beckman Coulter, Brea, CA, United States). 48 h after infection, dissociated HL-1 cells were resuspended in phosphate-buffered saline (PBS)-2 mM EDTA (Sigma-Aldrich). eGFP and mCherry expression were detected by excitation at 488 and 561 nm and detection at 525/40 and 610/20 nm, respectively.

### Confocal Imaging

A total of 24 h after seeding on glass coverslips, HL-1 cells were washed with PBS and fixed with 4% paraformaldehyde in PBS by incubation for 10 min at room temperature. Next, cells were treated for 10 min with 0.1% Tween 20 (Sigma-Aldrich) and 4’,6-diamidin-2 phenylindole (DAPI; Sigma-Aldrich; 1 μg/mL) to stain nuclei. Fluorescence images were taken using a confocal microscope (Nikon Eclipse TE300 equipped with Nikon C2 scanning head) using the Nikon CFI Plan Fluor 20× objective (Minato-ku, Tokyo, Japan). Excitation wavelengths of 355, 488 and 561 nm were used for DAPI, eGFP, and mCherry, respectively. Band-pass emission filters of 457/50, 528/38, and 620/15 nm were used for DAPI, eGFP, and mCherry, respectively. The confocal images consisted of 1024 × 1024 pixels.

### Patch-Clamp Recordings of APs on HL-1 Cells

Patch-clamp recordings were performed using the whole-cell configuration of the patch-clamp technique. The patch-clamp set-up has been described elsewhere (Sartiani et al., 2002a,b). Briefly, isolated cells were placed in an experimental bath on the platform of an inverted microscope (Nikon Diaphot TMD). Recordings were performed using a patch amplifier (Axopatch-200B, Axon Instruments, CA, United States) interfaced to a personal computer by means of a DAC/ADC interface (Labmaster Tekmar, Scientific Solutions, Hamilton, OH, United States). Data were viewed on-line on a computer screen. Experimental control, data acquisition and preliminary analysis were performed by means of the integrated software package pClamp (Axon Instruments). For action potential (AP) recordings, cells were superfused with normal Tyrode’s solution at room temperature. Patch-clamp pipettes, prepared from glass capillary tubes (Garner Glass, CA, United States) by means of a two-stage vertical puller (Hans Otchoski, Homburg, Germany), had a resistance of 3-4 MΩ when filled with the internal solution (composition (mM): K-Aspartate 130, Na_2_ATP 5, MgCl_2_ 2, CaCl_2_ 5, EGTA 11, HEPES–KOH 10, pH 7.2). The patch-clamped cell was superfused by means of a micro-superfusor, which allowed rapid changes of the solution bathing the cell. APs were elicited at 0.2 Hz in the current-clamp mode and sampled at 2 kHz.

### hiPSC Culture and Cardiac Differentiation

hiPSCs (WTC11) were differentiated by a monolayer-based protocol, using the cardiac PSC Cardiomyocyte Differentiation Kit (Life Technologies, Thermo Fisher Scientific, Carlsbad, CA, United States) and following the manufacturer’s instructions (Dell’Era et al., 2015; Pioner et al., 2019). Briefly, hiPSCs were maintained under feeder-free conditions in mTeSR Plus medium (Stem Cell Technologies, Vancouver) on a Matrigel hESC-Qualified Matrix (Corning, New York, NY, United States) and passaged every 4–5 days. For cardiac differentiation, 70–80% confluent hiPSC colonies were chemically dissociated using 1 × Tryple (Life Technologies). After suspension into mTeSR supplemented with 5μM ROCK inhibitor Y27632 (Stem Cell Technologies, 72302), cells were seeded as single cells onto Matrigel-coated 24 well plates at a density of 6x10^4 cells/well. At 70% confluency, which was reached after 2–3 days, the culture medium was changed to Cardiomyocyte Differentiation Medium A (referred as day 0) to start cardiomyogenic differentiation. After 2 days, medium A was replaced with Medium B, followed by Medium C after two additional days to promote final differentiation. Thereafter, cells were fed every other day with Medium C until the appearance of spontaneously beating monolayers, which occurred at day 8–10. At day 12, to complete hiPSC–CM maturation, Medium C was replaced with RPMI plus B27 supplement (Life Technologies, which was refreshed every 2 days. For AP recordings, hiPSC-CMs were used on day 30 of differentiation.

### MULTIPLE Optomechanical Design

**FIGURE 1:**
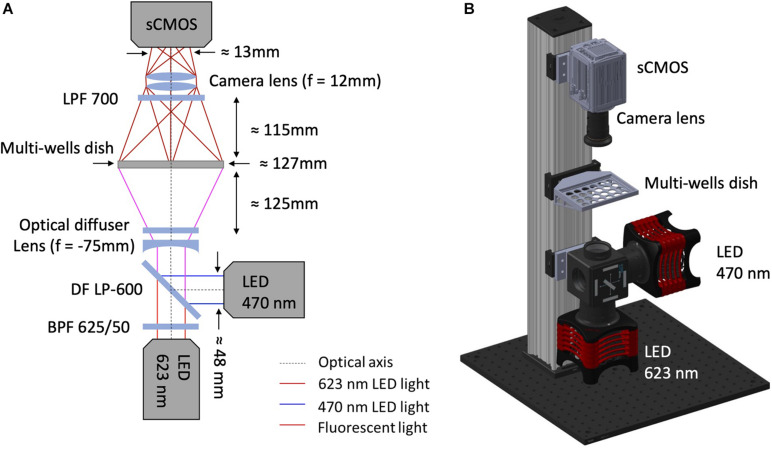
MULTIPLE optomechanical system design. **(A)** Optical scheme of MULTIPLE platform. The excitation system combines a red LED followed by a band-pass filter (625/50 nm) with a blue LED using a large-area dichroic beam mounted on a kinematic fluorescence cube (DFM2/M, Thorlabs). A divergent lens combined with an optical diffuser is used to homogenize the light intensity irradiating the multiwell plate. The fluorescence signal is filtered with a long-pass filter and focused into a sCMOS camera sensor through a camera lens. **(B)** 3D mechanical scheme of MULTIPLE characterized by a final footprint of 450 × 450 × 900 mm. The illumination, detection and sample holder blocks are vertically aligned on a construction rail through drop on rail carriages.

As shown in Figure 1, illumination for optical actuation and sensing is provided by high-power LEDs controlled by a LED driver (DC20, Thorlabs, Newton, NJ, United States). Optical actuation of ChR2 (H134R) and CheRiff is provided by a LED operating at a wavelength centered at 470 nm (SOLIS-470C, Thorlabs) while illumination for sensing is provided by a red LED centered at 623 nm (SOLIS-623C, Thorlabs), followed by a band-pass filter (625PB50, Omega Optical, Brattleboro, VT, United States). The light paths for optical sensing and actuation are combined by a large-area dichroic mirror (550 DCLP, Omega Optical) mounted on a kinematic fluorescence cube (DFM2/M, Thorlabs) holding also a plane-concave lens (f = –75 mm LC1315-A-ML, Thorlabs) and an optical diffuser (DG20-1500, Thorlabs). The diverging lens and the diffuser are exploited to achieve global and homogeneous illumination of the multiwell plate which is placed at ≈ 12 mm distance onto a customized 3D printed holder. The bottom part of the holder is designed to selectively deliver light to the wells thus avoiding spurious signals originating from the autofluorescence of the plastic culture plate. Emitted fluorescence is passed through a long-pass filter (LP700, Omega Optical) and collected in the forward direction by a camera lens (f = 12 mm, MVL12M43, Thorlabs) placed in front of an sCMOS camera (ORCA-Flash 4.0 V3, Hamamatsu Photonics, Hamamatsu City, Japan) operating at a frame rate of 100 Hz. The illumination, detection and sample holder blocks are vertically aligned on a construction rail (XT95-750, Thorlabs) through drop on rail carriages (XT95RC2/M, Thorlabs) for proper relative positioning between the blocks. Aluminum spacers produced by a traditional milling process were used to attach the illumination and detection blocks to the rail carriages at the optimal relative distance with respect to the vertical rail (Supplementary Figures 2, 3). All the commercial optomechanical components implemented in the MULTIPLE platform are listed in Table 1. The final overall dimensions of the MULTIPLE system are 450 × 450 × 900 mm. The SolidWorks file of the system is available upon request.

**TABLE 1:** MULTIPLE Optomechanical components list.

**Description**	**Code**	**Company**
Aluminum breadboard	MB4545/M	Thorlabs
900 mm long construction rail	XE25L900/M	Thorlabs
450 mm long construction rail	XE25L450/M	Thorlabs
95 mm construction rail	XT95-750	Thorlabs
Drop-on-rail carriage	XT95RC2/M	Thorlabs
12 mm fixed focal lengths	MVL12M43	Thorlabs
Kinematic fluorescence filter cube	DFM2/M	Thorlabs
High-power LED for microscopy	SOLIS-470C	Thorlabs
High-power LED for microscopy	SOLIS-623C	Thorlabs
Ø2” N-BK7 piano-concave lens	LC1315-A-ML	Thorlabs
High-power driver for solis LEDs	DC20	Thorlabs
Ø2” unmounted N-BK7 ground glass diffuser	DG20-1500	Thorlabs
sCMOS ORCA-Flash 4.0 V3	C13440-20CU	Hamamatsu Photonics
550 dichroic longpass	550DCLP	Omega Optical
625 nm bandpass 50 nm	625BP50	Omega Optical
700 nm longpass	700LP	Omega Optical
Base plate for 95 mm rails	XT95P3	Thorlabs
End plate for 95 mm construction Rails	XT95EC1	Thorlabs
Quick corner cube for 25 mm rails	XE25W3	Thorlabs

### Image and Data Analysis

The main experiments were carried out using a 24-well plate at the LEDs’ maximum irradiance. A custom-developed script in LabVIEW (National Instruments, Austin, TX, United States) was used to control the optical pacing blue LED in terms of pulse duration (10–50 ms) and frequency (1 Hz), to maintain the optical sensing LED switched on and to trigger the camera which was programmed to record from 5 to 10 s with 10-ms integration times through HC Image Live dedicated software (Hamamatsu Corporation, Sewickley, PA, United States).

After images recording, a second LabVIEW script was used to select a region of interest (ROI) for each well and to extract associated traces reported in terms of percent change of fluorescence from baseline (ΔF/F_0_). Raw traces were processed by photobleaching correction and normalization using Fiji-ImageJ (National Institutes of Health, Bethesda, MD) and OriginLab (Northampton, MA, United States) software. AO amplitude (APA) and duration (i.e., APD_50_ and APD_90_) were automatically extracted from normalized traces averaged over 9 beats.

## Results

### Illumination and Detection Performance of MULTIPLE

**FIGURE 2:**
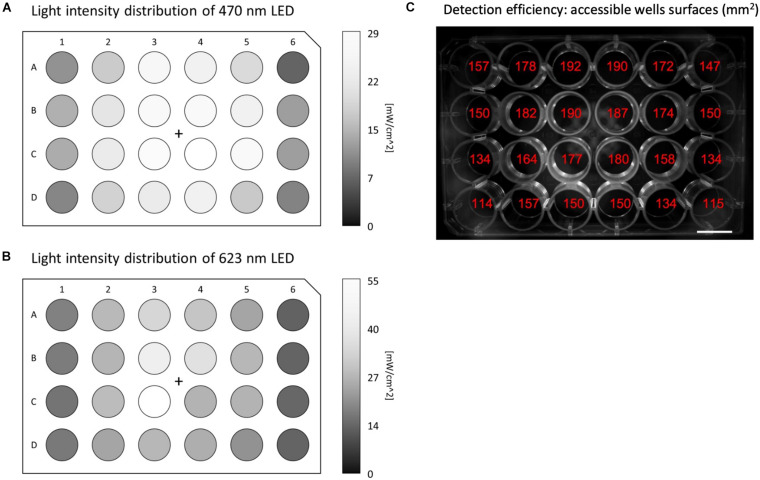
Illumination and detection performances of MULTIPLE. Light intensity distribution across the whole 24-well plate for **(A)** blue and **(B)** red LED light, respectively, using maximum LED power. The intensity of both LEDs radially decreases moving away from the optical axis causing a more than 50% reduction for the outermost wells. **(C)** Image of the 24-well plate reporting for each well the accessible area exploitable for ROI measurements. Detection losses registered at the peripheral wells are introduced by the non-telecentric camera adopted in the optical scheme.

MULTIPLE allows global illumination of multiwell plates by diverging a collimated beam produced by high-intensity LED matrices. The system combines two LED matrices emitting blue (for optogenetic actuation) and red (for AP sensing) light using a large-area dichroic beam splitter. The red LED matrix is filtered by a 2” band-pass filter to select the optimal spectral range for VSD excitation. Finally, an optical diffuser is placed in front of the divergent lens to homogenize the light intensity across the multiwell plate. This configuration allows a maximum light intensity on the multiwell plane along the optical axis of the order of 30 and 60 mW/cm^2^ for the blue and red light, respectively. As a consequence of this basic illumination scheme, the intensity radially decreases away from the optical axis causing non-uniform illumination across the multiwell plates (see Figures 2A,B). With the exception of the peripheral wells, we found an intensity reduction below 50% across the 24-well plate for both light sources. While the spatial heterogeneity of the red light intensity is not expected to be a critical issue (considering that potentiometric optical recordings imply normalized fluorescent signals), the heterogeneity of the blue light intensity could introduce interwell differences in the electrical response upon optical stimulation.

The fluorescence signal coming from the multiwell plate is collected in the forward direction using a camera lens place in front of a 4-megapixel sCMOS camera operating at a frame rate of 100 Hz. The fluorescence signal is filtered by a large-area long-pass filter placed in front of the camera lens. The non-telecentric camera lens adopted in this cost-effective optical scheme intrinsically introduces detection loss in the peripheral wells due to the perspective view of the multiwell plate (Figure 2C). However, by choosing a camera lens with an appropriate working distance this unwanted effect can be contained, and even in the outermost wells, signals can be collected from more than 100 mm^2^.

### Optical Recording of Optogenetically Induced APs in Channelrhodopsin-Expressing HL-1 Cells

**FIGURE 3:**
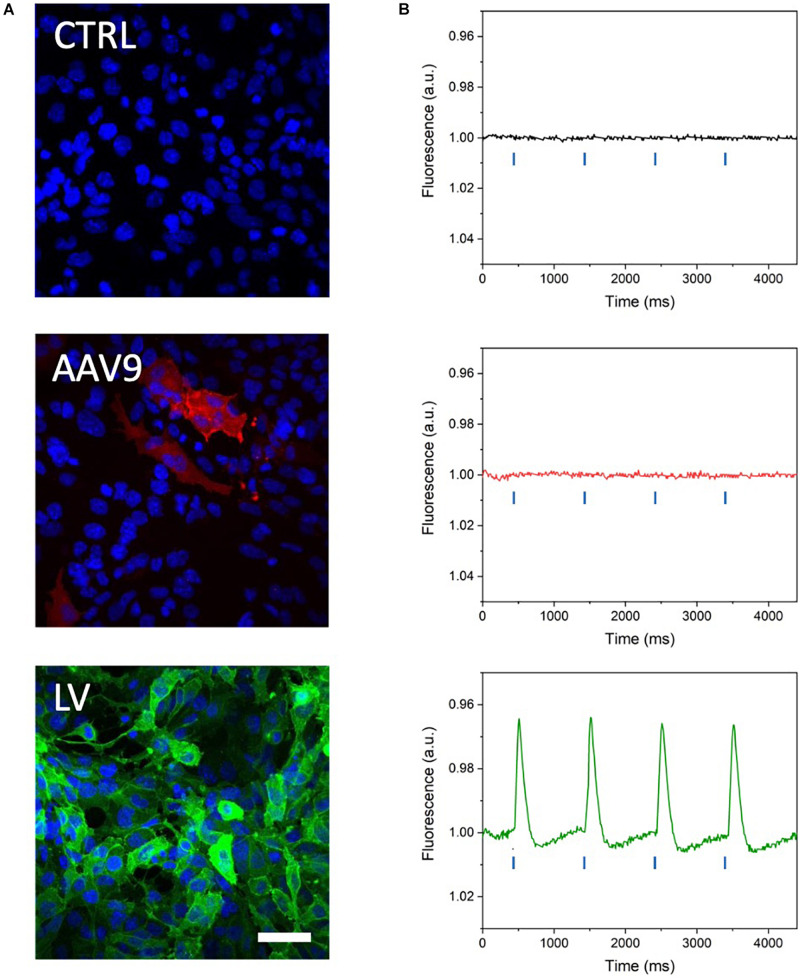
Optical recording of optogenetically induced APs in channelrhodopsin-expressing HL-1 cells. **(A)** Representative confocal microscope images of HL-1 cells expressing blue light-activable ion channels after transduction with a commercial AAV serotype capsid 9-pseudotyped AAVV encoding mCherry-tagged ChR2(H134R) (red channel) and a custom-made vesicular stomatitis G protein-pseudotyped LV encoding eGFP-tagged CheRiff expression (green channel). Cell nuclei were stained with DAPI (blue channel). The higher eGFP signal attests to the more efficient transduction of the HL-1 cells with the LV than with the AAVV. Scale bar = 25 μm. **(B)** Representative traces of optically induced APs registered in AAVV- and LV-transduced HL-1 cell layers after loading of the cells with VSD and stimulating with blue light pulses at 1 Hz (blue lines in the graph). Consistent with the higher transduction efficiency of the HL-1 cells, APs were registered only in the LV-transduced HL-1 cells. CTRL, untransduced HL-1 cells.

**FIGURE 4:**
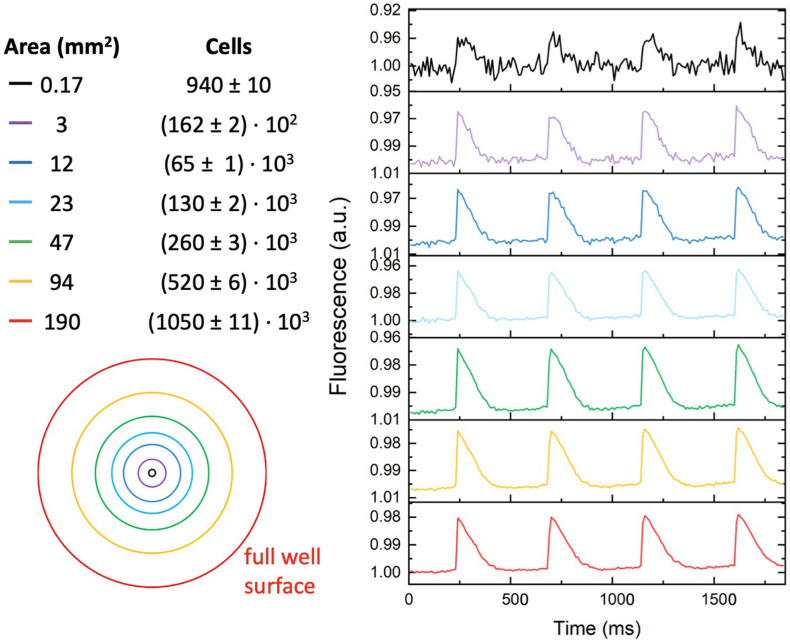
Signal-to-noise ratio *versus* detection area. Representative optical recording of optogenetically induced APs collected from different-sized surface areas (2 Hz stimulation frequency with blue light pulses duration of 30 ms). Reduction of the detection area from the whole well area (≈190 mm^2^) to 0.17 mm^2^, reduced the signal-to-noise ratio from ≈ 600 to ≈ 2. The estimated number of cells present in the different-sized detected area is indicated in the right column.

MULTIPLE has been tested on mouse atrial myocyte-like cells (i.e., HL-1 cells) expressing blue light-activatable cation channels. To this end, HL-1 cells were either transduced with an AAVV encoding mCherry-tagged ChR2(H134R) or with an LV coding for eGFP-tagged CheRiff. The transduced cell populations were analyzed by flow cytometry, which resulted in the detection of, respectively, 51.74% mCherry^+^ cells and 97.52% eGFP^+^ cells (Supplementary Figure 1). Confocal imaging of the transduced HL-1 cell populations confirmed the results of the flow cytometric analysis by showing transduction of nearly all cells with the LV, but only a subpopulation of the cells using the AAVV (Figure 3A). By loading cell cultures with a near-infrared VSD (i.e., di-4-ANBDQPQ), we assessed the capability of MULTIPLE in combination with optogenetically induced APs. Cells were used at 100% confluency corresponding to (5.53 ± 0.06).10^5^ cells/cm^2^ in each well. This initial investigation was performed on wells placed close to the optical axis of the MULTIPLE system for achieving maximum light intensities. During channelrhodopsin activation using 30-ms blue light pulses (stimulation frequency of 1 Hz), the cells were constantly illuminated with red light for optical detection of APs. As shown in Figure 3B, under these conditions, we were able to detect optically induced APs only in cells transduced with the LV. This result shows that under proper illumination conditions, MULTIPLE allows to optically induce and record APs in HL-1 cells stably expressing CheRiff. This investigation was performed integrating the signal derived from entire wells (190 mm^2^). However, MULTIPLE sensitivity allows to detect APs also in a significantly smaller area (Figure 4). Setting a signal-to-noise ratio 2 as limit of detection, we were able to detect APs in areas as small as of 0.17 mm^2^, which corresponds of 10^3^ cells.

### AP Kinetics vs. Optogenetic Pulse Duration

**FIGURE 5:**
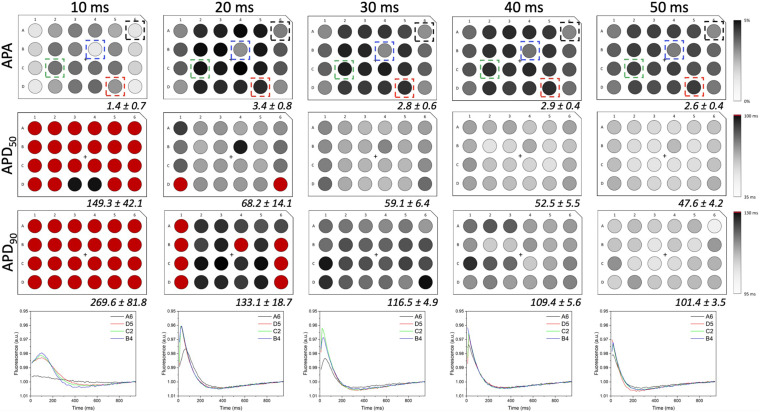
AP kinetics *versus* optogenetic pulse duration. APA and AP duration (i.e., APD_50_ and APD_90_) values registered from LV-transduced HL-1 cells seeded in 24-well plates and irradiated at maximum light intensities and for the pulse durations indicated at the top of the figure (1 Hz stimulation frequency). Average ± standard deviation of APAs and AP durations were calculated for all 24 wells in the plate and for each experimental condition. Representative traces taken from wells placed at different distances from the optical axis are shown for each stimulation time. By increasing pulses duration from 10 to 50 ms, interwell differences decreased for all the AP parameters considered, thus overcoming the spatial heterogeneity induced by the simplicity of the implemented optical scheme.

**FIGURE 6:**
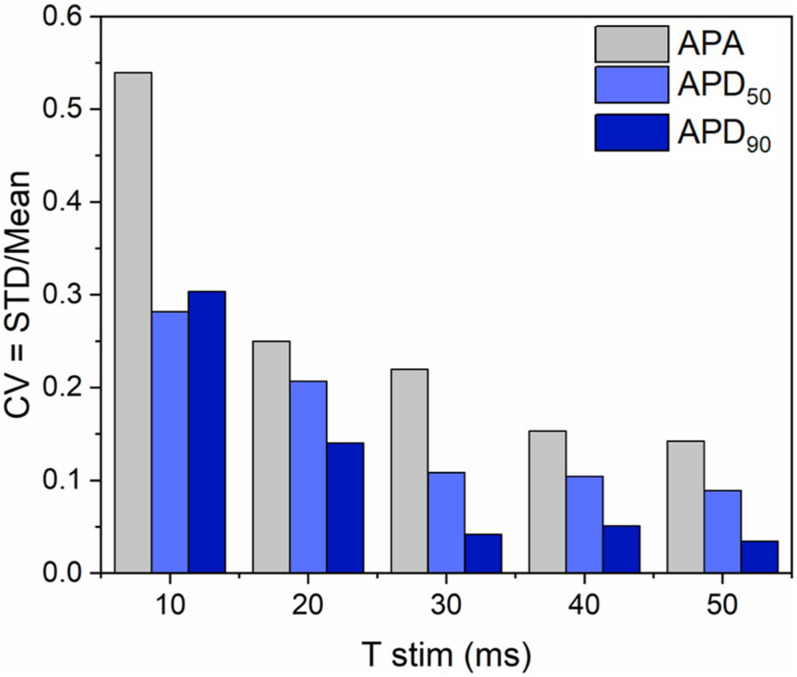
Interwell variability of optogenetically induced APs. Interwell variability of APA and AP duration as a function of the light pulse duration. Upon increasing the optical stimulus from 10 to 50 ms, decreasing trends in the interwell variability of APA and AP duration (i.e., APD_50_ and APD_90_) were observed, confirming that differences in AP triggering due to non-uniform illumination can be overcome by stimulating with pulses ≥ 30 ms.

**FIGURE 7:**
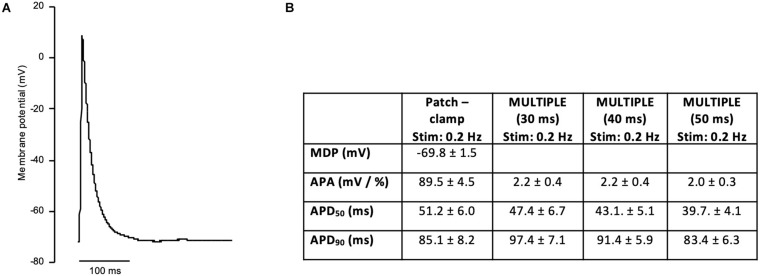
Patch-clamp recording of AP in HL-1 cells. **(A)** Representative AP recording on a HL-1 cells using single cell patch-clamp (whole-cell configuration). APs were elicited at 0.2 Hz in current-clamp mode. **(B)** Table reporting average ± standard error of maximal diastolic potential (MDP), APA, APD_50_, and APD_90_ measured in patch-clamp cells (number of cells = 15). APA, APD_50_, and APD_90_ measured with MULTIPLE (pulses duration of 30, 40, and 50 ms) at the same stimulation frequency (0.2 Hz) are reported for direct comparison.

In order to investigate if the non-uniform blue light intensity distribution affects the interwell AP kinetics, CheRiff-expressing HL-1 cells were irradiated at maximum light intensities while being stimulated with light pulses of increasing duration from 10 to 50 ms (at 1 Hz stimulation frequency; Figure 5). As expected, significant interwell differences in APA, APD_50_, and APD_90_ were found, especially for pulse duration lower than 30 ms. This heterogeneity is probably related to the fact that CheRiff depolarizing current is not sufficient to activate the whole cell population producing an overall reduction of APA as well as an increment of APD probably ascribable to currents propagation across the electrically coupled confluent cells. It is worth reminding that the main repolarizing current in these cells is I_*Kr*_, which undergoes rapid C-type inactivation upon depolarization thus reducing the net outward current and delaying repolarization. For pulse duration higher than 30 ms, lower interwell variability of AP kinetics was observed as evinced by smaller standard deviations for APA, APD_50_, and APD_90_ across wells. The coefficient of variation (CV) calculated for the three AP dynamic parameters, corroborated the trend of decreasing interwell variability in AP kinetics with increasing optical pulse duration (Figure 6). This identifies 30 ms as the pulse duration threshold needed in MULTIPLE for proper optogenetic induction of APs across the whole plate. The need to apply relatively long stimulation pulses to elicit APs relates to the low blue light intensity achievable with our optical scheme considering, which is one order of magnitude lower than the blue light intensity typically used for optogenetic stimulation of cardiomyocytes (∼ mW/mm^2^) (Crocini et al., 2016; Scardigli et al., 2018). This is nicely illustrated by an experiment in which we raised the blue light intensity by removing the divergence lens to concentrate the blue LED only on the central portion of the multi-well plate. As expected, increasing the light intensity allowed us to optogenetically induce APs using stimulation pulses much shorter than 30 ms (Supplementary Figure 4). Finally, to check whether long optogenetic stimulation pulses may affect repolarization, optical recording of optogenetically induced APs (pulses duration: 30, 40, and 50 ms at 0.2 Hz stimulation frequency) were directly compared with single cell patch-clamp recording where APs were induced by inward current injection (2 ms current square pulses, 500–1000 pA, at 0.2 Hz frequency; Figure 7). Notably, no differences were found in ADP_50_ and APD_90_ recorded by means of the two approaches, thus demonstrating MULTIPLE’s suitability in assessing AP dynamics. Based on these findings, all subsequent measurements were performed at maximum light intensities and by using 40-ms blue light pulses.

### MULTIPLE Detection of AP Duration With E-4031

**FIGURE 8:**
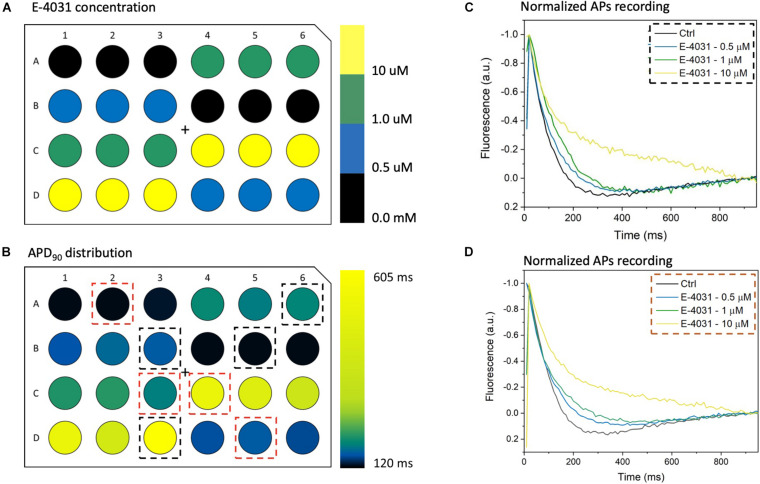
MULTIPLE detection of AP duration with E-4031. **(A)** E-4031, a selective Kv11.1 channel blocker, was applied at 7 different concentrations ranging from 0 to 10 μM to CheRiff-expressing HL-1 cells in a 24-well plate with each dose given to six wells distributed across the whole plate. **(B)** Resulting APD_90_ values were quantified, which attested that interwell variability was not a critical issue since similar values were registered from wells with cells exposed to the same E-4031 concentration. **(C,D)** Average AP traces of CheRiff-expressing HL-1 cells from 10 optical recordings (1 Hz stimulation frequency). For comparison datasets (two for each concentration) were taken from wells randomly positioned. AP duration increased in a drug dose-dependent manner, with clear APD prolongation at E-4031 concentrations higher than 1 μM.

**FIGURE 9:**
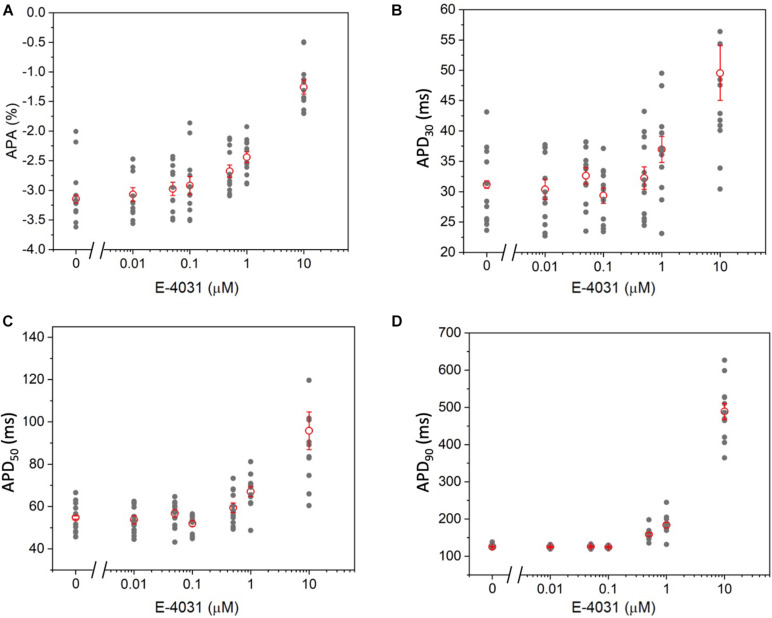
Dose–dependent effects of E-4031 on AP kinetics. **(A)** APA, **(B)** APD_30_, **(C)** APD_50_, and **(D)** APD_90_ as a function of E-4031 dose.

To further validate the high-throughput capability of MULTIPLE as well as to illustrate the range of dynamic information that can be acquired simultaneously, a dose response experiment was performed using the selective K_*v*_11.1 channel blocker E-4031. The drug was applied in 7 doses (0 – 10 μM) to CheRiff-expressing HL-1 layers across the entire 24-well plate according to the scheme shown in Figure 8A. This specific drug loading configuration was selected to further demonstrate that, by using optimal acquisition parameters in terms of light intensity and pulse duration, undesired effects of optical pacing due to non-uniform illumination can be overcome. Indeed, as clearly depicted in Figures 8B–D very similar APD90 values were obtained for wells exposed to the same concentration of E-4031, irrespective of their position in the culture plate. However, randomization in assigning wells with same drug concentration could be beneficial to improve the solidness of data acquired during a parallel investigation. As expected, prolongation of AP duration was observed especially at drug concentrations above 0.5 μM due to E-4031 inhibiting the rapidly activating delayed rectifier K^+^ current (I_*Kr*_) (Figures 8C,D). Thus, MULTIPLE was able to successfully track the dose-dependent effects of E-4031 in terms of AP duration even at lower concentrations of this class III antiarrhythmic drug (Figure 9). The effect of E4031 on AP duration is in line with that observed previously in Hl-1 cells (Wondergem et al., 2012) and in mouse native atrial cardiomyocytes (Nakamura et al., 2010). Interestingly, the decrease of AP amplitude induced by E-4031 has already been reported in HL-1 cells (Wondergem et al., 2012) and likely attributable to depolarization of membrane potential with consequent reduction of inward currents, I_*Na*_ and I_*Ca,T*_. Further investigation is necessary to fully clarify this point.

### Detection and Modulation of Spontaneous APs on hiPSC-CMs

**FIGURE 10:**
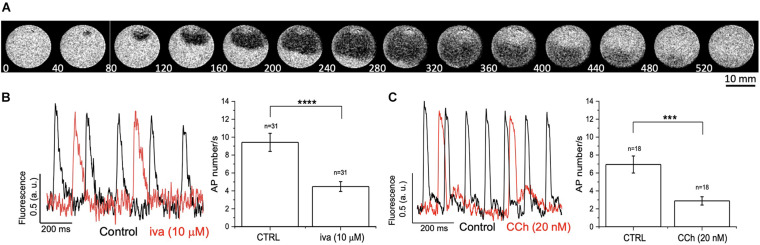
Effect of ivabradine and carbachol on spontaneously beating hiPSC-CMs. **(A)** Representative fluorescence imaging of spontaneous AP propagation in a hiPSC-CM monolayer during a 520 ms recording. **(B,C)** Spontaneous AP traces recorded in hiPSC-CM monolayer in the absence (black lines, CTR) and presence (red lines) of ivabradine (Iva, 10 μM) or carbachol (CCh, 20 nM). Histograms represent AP number/s, expressed as mean ± standard error of the mean, before and after Iva or CCh treatment. ****p < 0.0001 CTRL vs. Iva (10 μM); ***p < 0.001 CTRL vs. CCh (20 nM).

As a final experiment, we tested MULTIPLE’s ability to detect APs in monolayer of hiPSC-CMs at day 30 of differentiation. In this developmental stage, hiPSC-CMs display spontaneous firing activity that retains major functional properties of human native pacemaker centers and respond to heart rate-limiting drugs (Blazeski et al., 2012; Mandel et al., 2012; Karakikes et al., 2015). hiPSC-CMs were loaded with di-4-ANBDQPQ and excited with red LED light in order to detect variations of membrane potential arising from spontaneous electrical activity. This allowed detection of repetitive membrane voltage variations that typically originated from clustered cells and propagated throughout the monolayer (Figure 10A). Next, in order to validate MULTIPLE ability to detect pharmacological responses in the hiPSC-CM model, we challenged the system with two different agents, namely ivabradine and carbachol, which are able to negatively modulate native human pacemaker activity by targeting hyperpolarization-activated cyclic nucleotide gated (HCN) channels and muscarinic receptors, respectively. Figure 10B shows representative traces of APs detected from hiPSC-CMs before and after application of ivabradine (10μM, Figure 10B) or carbachol (20 nM, Figure 10C), which in both cases decreased spontaneous AP firing rate, as expected.

## Discussion

### Advantages, Limitations, and Optical Implementations

MULTIPLE represents a simple and cost-effective optical platform engineered to enable fast characterization of cardiac electrophysiology. To this end, standard optical technologies to simultaneously control and monitor AP features of cells in multiwell plates are integrated in a compact platform. The main advantage of MULTIPLE is its high-speed investigation capacity achieved by coupling a scientific CMOS sensor with a camera lens. The large field of view enables parallel detection of the optical signal coming from the entire plate thus overcoming limitations of state-of-the-art optical systems, which commonly rely on well-by-well serial analysis. The simplicity of the optical scheme, as well as the cost-effectiveness of MULTIPLE, are further reflected in the illumination path where two high-power LED arrays are used to globally illuminate the multiwell plate enabling simultaneous voltage imaging and optogenetic stimulation. The practical applicability of MULTIPLE was demonstrated by probing the AP-modulating effects of a K_*v*_11.1 channel blocker at different concentrations during a single imaging session and by investigating the responses of hiPSC-AMs to chronotropic agents.

Despite its advantages, MULTIPLE has some limitations. The biggest limitation of MULTIPLE is the low and spatially heterogeneous light intensity especially with respect to the blue light. As shown in the Result section, the non-uniform illumination affects the efficiency of optical stimulation, especially for the peripheral wells. In the present work, we show that application of blue light-pulse durations longer than 30 ms activates all the wells with AP kinetics similar to those observed by electrical recordings. However, we cannot rule out the possibility that long-lasting and/or high-frequency optogenetic stimulation of the cells with long blue light pulses alters their electrophysiological responses. Considering that this effect could significantly depend on the cellular type, an *ad hoc* investigation is recommended based on the specific MULTIPLE employment. Another limitation of the proposed platform is that it is not telecentric. Although we showed that this aspect does not represent a true limiting factor in a 24-well plate, this may become problematic using plates with a higher number of wells.

Initially designed as an inexpensive and easy to assemble platform, the performance of MULTIPLE could be improved by adopting specialized optics, more powerful light sources and faster detectors. For example, a more sophisticated illumination scheme based on high-power LEDs and projection lens could be employed to maximize and homogenize the light intensity across the multiwell. Moreover, orthographic collection of the fluorescent signal using a high-resolution telecentric lens would be very beneficial in increasing the detection efficiency from all wells. Finally, the system could be equipped with a next-generation CMOS sensor capable of reaching kHz frame rates in a full-frame configuration, which would be advantageous for recording the fast up-stroke phase of cardiac APs.

### Applicability

Screening compound libraries of drug candidates is expensive, time-consuming and usually involves extensive use of animal models. To ameliorate cost effectiveness ratio, predictive *in vitro* models of cell monolayers in multiwell plates are increasingly being utilized for drug screenings because of their simplicity and the low consumption of candidate compounds and target cells. In the field of excitable cells, such as cardiomyocytes or neurons, fast and reliable *in vitro* approaches to study drugs modulating ion channel activity in multicellular preparations are particularly appealing but are still poorly available among current experimental sources. MULTIPLE fills at least part of this gap, providing a screening tool able to detect cardiac AP profiles of cell monolayers in 24-well plates. Importantly, using MULTIPLE, the assessment of the acute effects of a drug on the cardiac AP was rapid and accurate, which provides a great advantage compared to classical single cell-recordings by the patch-clamp technique. Indeed, MULTIPLE allowed fast investigation of drugs effect in HL-1 cells as demonstrated employing the selective K_*v*_11.1 channel blocker (E-4031), in a dose titration experiment. Furthermore, being designed for use in combination with multiwell plates, MULTIPLE allows parallel testing of various compounds and/or of a single compound at different concentrations, reducing the variability due to use of different cell batches or other experimental variables usually present in subsequent experimental sessions. As proof of principle MULTIPLE has been also used to assess electrical dynamic in spontaneously beating hiPSC-CM. The sensitivity of the platform allows to monitor wave propagation within a single well thus opening the possibility to test the effects of drug on conduction velocity. Finally, we validated MULTIPLE’s ability to detect pharmacological responses in the hiPSC-CM model using ivabradine and carbachol, which both gave expected effect on spontaneous electrical activity. This finding further extends and corroborates MULTIPLE potential as valuable experimental approach to study the effect of drugs on cardiomyocyte electrical properties.

Based on the studies described above, future applications will explore the potential of MULTIPLE to detect mid- and long-term effects of biologically relevant compounds in the hiPSC-CM model. To this end, MULTIPLE will be equipped with a heating system and O_2_/CO_2_ regulation, which are crucial to preserve the excitability of living cells in long-term experiments. Furthermore, future investigations will explore whether MULTIPLE can be used in combination with other cell types, including c smooth muscle cells and neurons differentiated from hiPSCs. All these cell models are readily amenable to genetic modification and at present represent appealing platforms of relevant translation value for preclinical screening and safety studies of novel drug candidates.

## Data Availability Statement

The original contributions presented in the study are included in the article/Supplementary Material, further inquiries can be directed to the corresponding author.

## Author Contributions

LSac, CCr, LSar, and EC contributed to the conception and design of the study. CCi, LSac, and CCr developed and characterized the optical system. LSac and SB developed the software. AV produced the lentivirus. LL syntetized the VSD. FP contributed with equipment. SB, VB, and UM performed the transfection. SB, VB, LSac, and CCr performed the optical measurements. CCr and LSac performed the analysis. LSac, VB, and CCr wrote the first draft of the manuscript. All authors contributed to manuscript revision, read, and approved the submitted version.

## Conflict of Interest

The authors declare that the research was conducted in the absence of any commercial or financial relationships that could be construed as a potential conflict of interest.

## Publisher’s Note

All claims expressed in this article are solely those of the authors and do not necessarily represent those of their affiliated organizations, or those of the publisher, the editors and the reviewers. Any product that may be evaluated in this article, or claim that may be made by its manufacturer, is not guaranteed or endorsed by the publisher.

## References

[B1] Ashraf M. Mohanan S. Sim B. R. Tam A. Rahemipour K. Brousseau D. (2021). Random access parallel microscopy. *Elife* 10:e56426. 10.7554/eLife.56426 33432922 PMC7843131

[B2] Blazeski A. Zhu R. Hunter D. W. Weinberg S. H. Zambidis E. T. Tung L. (2012). Cardiomyocytes derived from human induced pluripotent stem cells as models for normal and diseased cardiac electrophysiology and contractility. *Prog. Biophys. Mol. Biol.* 110 166–177. 10.1016/j.pbiomolbio.2012.07.013 22971665 PMC3910285

[B3] Bruegmann T. Malan D. Hesse M. Beiert T. Fuegemann C. J. Fleischmann B. K. (2010). Optogenetic control of heart muscle in vitro and in vivo. *Nat. Methods* 7 897–900. 10.1038/nmeth.1512 20881965

[B4] Claycomb W. C. Lanson N. A. Jr. Stallworth B. S. Egeland D. B. Delcarpio J. B. Bahinski A. (1998). HL-1 cells: a cardiac muscle cell line that contracts and retains phenotypic characteristics of the adult cardiomyocyte. *Proc. Natl. Acad. Sci. U.S.A.* 95 2979–2984. 10.1073/pnas.95.6.2979 9501201 PMC19680

[B5] Crocini C. Ferrantini C. Coppini R. Scardigli M. Yan P. Loew L. M. (2016). Optogenetics design of mechanistically-based stimulation patterns for cardiac defibrillation. *Sci. Rep.* 17:35628. 10.1038/srep35628 27748433 PMC5066272

[B6] Dell’Era P. Benzoni P. Crescini E. Valle M. Xia E. Consiglio A. (2015). Cardiac disease modeling using induced pluripotent stem cell-derived human cardiomyocytes. *World J. Stem Cells* 7 329–342. 10.4252/wjsc.v7.i2.329 25815118 PMC4369490

[B7] Dunlop J. Bowlby M. Peri R. Vasilyev D. Arias R. (2008). High-throughput electrophysiology: an emerging paradigm for ion-channel screening and physiology. *Nat. Rev. Drug Discov.* 7 358–368. 10.1038/nrd2552 18356919

[B8] Hansen A. Eder A. Bönstrup M. Flato M. Mewe M. Schaaf S. (2010). Development of a drug screening platform based on engineered heart tissue. *Circ. Res.* 107:35. 10.1161/CIRCRESAHA.109.211458 20448218

[B9] Hochbaum D. Zhao Y. Farhi S. Klapoetke N. Werley C. A. Kapoor V. (2014). All-optical electrophysiology in mammalian neurons using engineered microbial rhodopsins. *Nat. Methods* 11 825–833. 10.1038/nmeth.3000 24952910 PMC4117813

[B10] Karakikes I. Ameen M. Termglinchan V. Wu J. C. (2015). Human induced pluripotent stem cell-derived cardiomyocytes: insights into molecular, cellular, and functional phenotypes. *Circ. Res.* 117 80–88. 10.1161/circresaha.117.305365 26089365 PMC4546707

[B11] Klimas A. Ambrosi C. Yu J. Williams J. C. Bien H. Entcheva E. (2016). OptoDyCE as an automated system for high-throughput all-optical dynamic cardiac electrophysiology. *Nat. Commun.* 7:11542. 10.1038/ncomms11542 27161419 PMC4866323

[B12] Mandel Y. Weissman A. Schick R. Barad L. Novak A. Meiry G. (2012). Human embryonic and induced pluripotent stem cell-derived cardiomyocytes exhibit beat rate variability and power-law behavior. *Circulation* 125 883–893. 10.1161/circulationaha.111.045146 22261196 PMC3697086

[B13] Matiukas A. Mitrea B. G. Qin M. Pertsov A. M. Shvedko A. G. Warren M. D. (2007). Near-infrared voltage-sensitive fluorescent dyes optimized for optical mapping in blood-perfused myocardium. *Heart Rhythm.* 4:1441. 10.1016/j.hrthm.2007.07.012 17954405 PMC2121222

[B14] McGlynn K. P. LaChaud Q. Burton F. L. Smith G. L. (2018). Measurements of action potentials, intracellular Ca2+ and contraction in stimulated isolated adult cardiomyocytes using CellOPTIQ^®^ platform for lead development screening. *J. Pharmacol. Toxicol. Methods* 93:119. 10.1016/j.vascn.2018.01.406

[B15] Nakamura H. Ding W. Sanada M. Maeda K. Kawai H. Maegawa H. (2010). Presence and functional role of the rapidly activating delayed rectifier K(+) current in left and right atria of adult mice. *Eur. J. Pharmacol.* 649 14–22. 10.1016/j.ejphar.2010.08.025 20826138

[B16] Pioner J. M. Santini L. Palandri C. Martella D. Lupi F. Langione M. (2019). Optical investigation of action potential and calcium handling maturation of hiPSC-cardiomyocytes on biomimetic substrates. *Int. J. Mol. Sci.* 20:3799. 10.3390/ijms20153799 31382622 PMC6695920

[B17] Sartiani L. Bochet P. Cerbai E. Mugelli A. Fischmeister R. (2002a). Functional expression of the hyperpolarization-activated, non-selective cation current I(f) in immortalized HL-1 cardiomyocytes. *J. Physiol.* 545 81–92. 10.1113/jphysiol.2002.021535 12433951 PMC2290645

[B18] Sartiani L. Cerbai E. Lonardo G. DePaoli P. Tattoli M. Cagiano R. (2002b). Prenatal exposure to carbon monoxide affects postnatal cellular electrophysiological maturation of the rat heart: a potential substrate for arrhythmogenesis in infancy. *Circulation* 109 419–423. 10.1161/01.CIR.0000109497.73223.4D 14718404

[B19] Scardigli M. Müllenbroich C. Margoni E. Cannazzaro S. Crocini C. Ferrantini C. (2018). Real-time optical manipulation of cardiac conduction in intact hearts. *J. Physiol.* 596 3841–3858. 10.1113/JP276283 29989169 PMC6117584

[B20] Wondergem R. Graves B. M. Li C. Williams D. L. (2012). Lipopolysaccharide prolongs action potential duration in HL-1 mouse cardiomyocytes. *Am. J. Physiol. Cell Physiol.* 303 C825–C833. 10.1152/ajpcell.00173.2012 22895260 PMC3469715

