# Supplementary material for: Fast Optical Investigation of Cardiac Electrophysiology by Parallel Detection in Multiwell Plates
Source: Front Physiol. 2021 Sep 3;12:692496. doi: 10.3389/fphys.2021.692496 (PMC8446431; doi:10.3389/fphys.2021.692496)
Supplement: Supplementary file 1 [file Data_Sheet_1.pdf]

Supplementary Material

Supplementary Figures

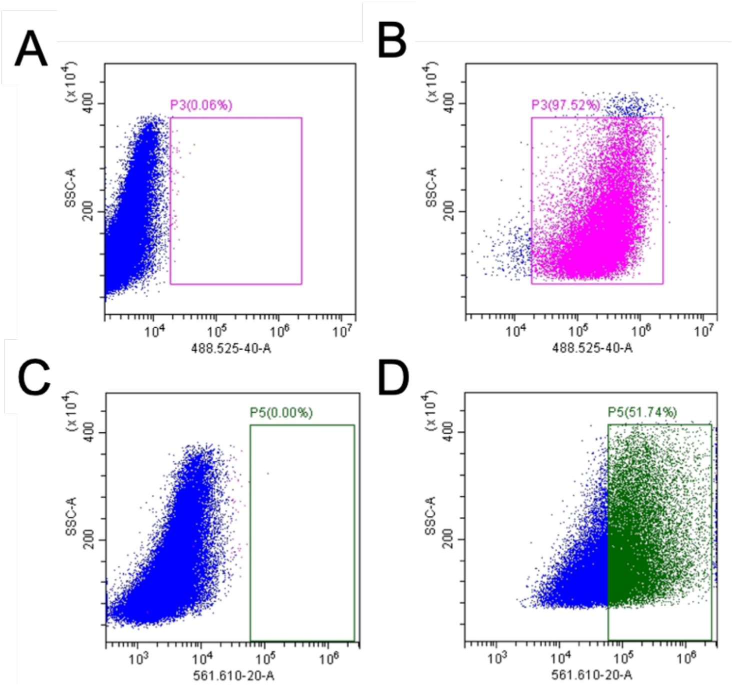

Supplementary Figure 1: Flow cytometric analysis of channelrhodopsin expression in HL-1 cells. Transduction of HL-1 cells with the CheRiff-encoding LV or the ChR2(H134R)-encoding AAVV resulted in 97.52% $eGFP^{+}$ (B) and 51.74% $mCherry^{+}$ (D) HL-1 cells, respectively, *versus* 0.06% (A) and 0.00% (C) of positive hits under control conditions.

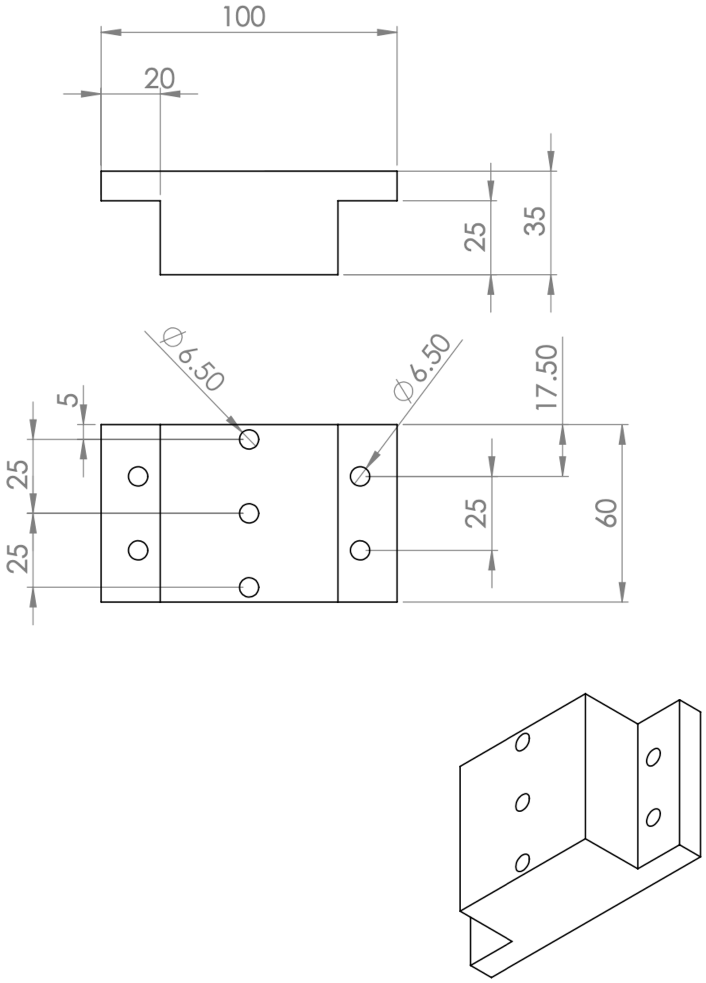

Figure S2: Camera holder technical drawing.

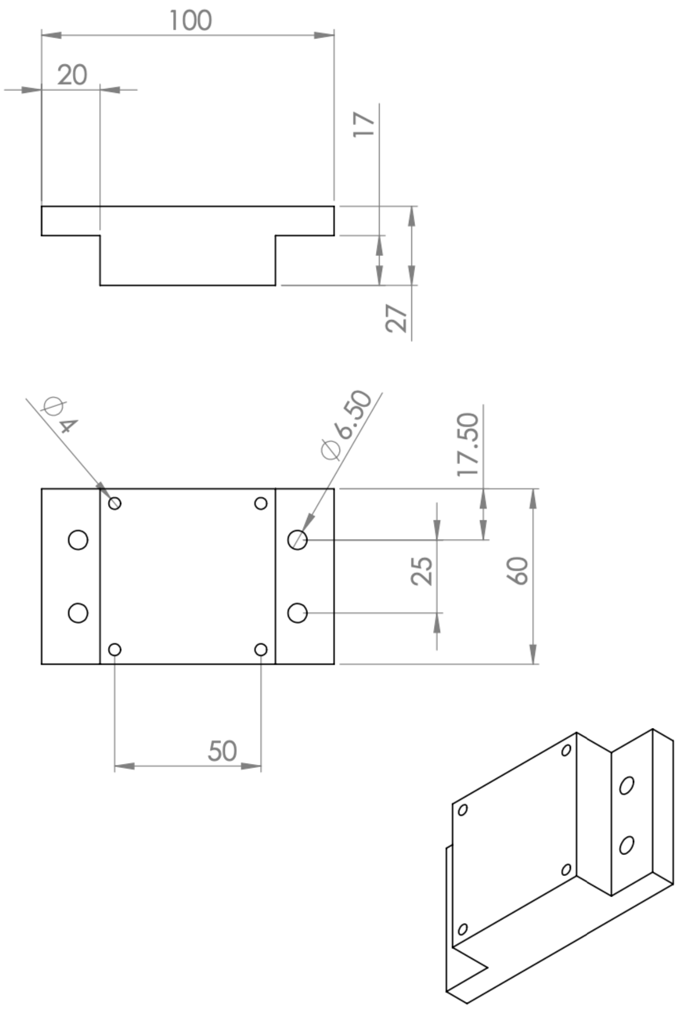

Figure S3: Cube holder technical drawing.

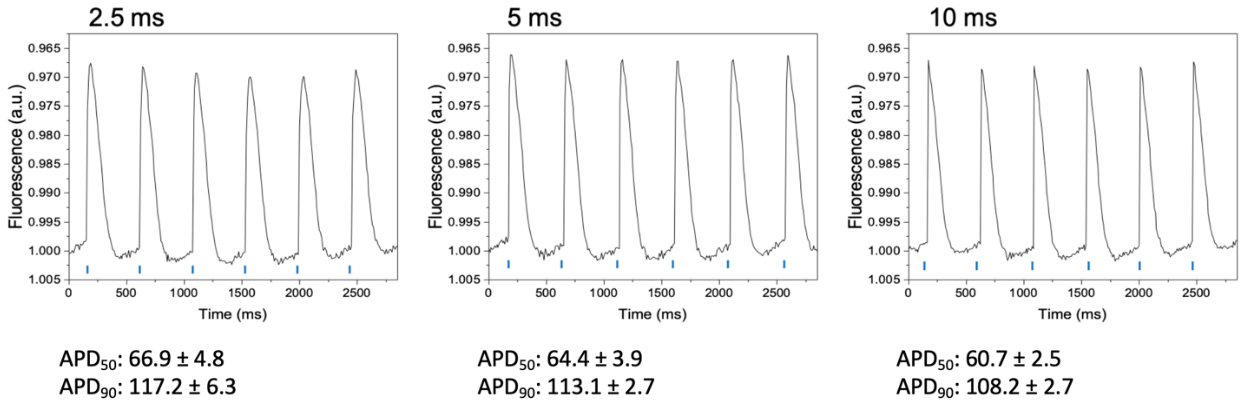

Figure S4: Optical recording of optogenetically induced APs in channelrhodopsin-expressing HL-1 cells using short blue light pulses. Representative traces of APs elicited by applying a blue light pulse of 2.5, 5 or 10 ms. In this proof of principle experiment, the blue light intensity was raised by removing the divergence lens after the LED matrices. This allows stimulating the central portion of the multi-well plate (approximately four wells) with a blue light intensity of the order of 2 mW/mm^2. Red light intensity was set at the same value used in the experiment described in the main text. APD_{50} and APD_{90} are reported for each pulse length on the average of four wells.
