# Supplementary material for: Fast Optical Investigation of Cardiac Electrophysiology by Parallel Detection in Multiwell Plates
Source: Front Physiol. 2021 Sep 3;12:692496. doi: 10.3389/fphys.2021.692496 (PMC8446431; doi:10.3389/fphys.2021.692496)
Supplement: Supplementary file 2 [file Table_1.docx]

| **Description** | **Company** | **Code** |
| --- | --- | --- |
| Aluminium Breadboard | Thorlabs | MB4545/M |
| 95 mm Construction Rail | Thorlabs | XT95-750 |
| Base Plate for 95 mm Rail | Thorlabs | XT95P3 |
| Drop-On-Rail Carriage | Thorlabs | XT95RC2/M |
| Kinematic Fluorescence Filter Cube | Thorlabs | DFM2/M |
| 550 Dichroic Long-pass | Omega Optical | 550DCLP |
| 625 nm Bandpass 50 nm | Omega Optical | 625BP50 |
| High-Power LED 623 nm | Thorlabs | SOLIS-623C |
| High-Power LED 470 nm | Thorlabs | SOLIS-470C |
| Plano-Concave Lens | Thorlabs | LC1315-A-ML |
| Diffuser | Thorlabs | DG20-1500 |
| sCMOS camera (ORCA-Flash 4.0 V3) | Hamamatsu | C13440-20CU |
| 700 nm Long-pass | Omega Optical | 700LP |
| Camera lens | Thorlabs | MVL12M43 |
